# Photochromic Sensors for Paper Marking

**DOI:** 10.3390/ma18112501

**Published:** 2025-05-26

**Authors:** Elżbieta Sąsiadek-Andrzejczak, Malwina Jaszczak-Kuligowska, Mariusz Dudek, Adam K. Puszkarz, Marek Kozicki

**Affiliations:** 1 Department of Mechanical Engineering, Informatics and Chemistry of Polymer Materials, Faculty of Materials Technologies and Textile Design, Lodz University of Technology, Żeromskiego 116, 90-543 Lodz, Poland; malwina.jaszczak@p.lodz.pl; 2 Institute of Materials Science and Engineering, Lodz University of Technology, 90-537 Lodz, Poland; mariusz.dudek@p.lodz.pl; 3 Textile Institute, Faculty of Materials Technologies and Textile Design, Lodz University of Technology, Żeromskiego 116, 90-543 Lodz, Poland; adam.puszkarz@p.lodz.pl

**Keywords:** photochromic sensor, UV radiation sensor, paper marking, paper securing, security element, UV retarder

## Abstract

This study presents UV radiation sensors for use as paper marking. The sensors turn pink under exposure to UVA radiation and the color change is reversible. Additionally, a UV radiation retarder was applied to the sensor to delay the reaction and weaken the change in sensor color. The color changes of the sensors were analyzed depending on the absorbed dose of UVA radiation using reflectance spectrophotometry. Furthermore, the chemical analysis and surface morphology of the samples were performed using Raman Spectroscopy and Scanning Electron Microscopy, respectively. In addition, the structure of the sensors on the paper surface was assessed using X-ray Micro-Computed Tomography. Finally, possible potential applications for these types of sensors were presented, including marking, securing, and protecting against the counterfeiting of documents, paper packaging, and other paper products, and creating decorative elements, as well as measuring the 2D/3D dose distribution of UV radiation on paper products.

## 1. Introduction

Various methods of protection against the counterfeiting of paper-based materials, such as documents or packaging, are used, which can be classified into three categories: overt, certified, and hidden protections. Overt protections are a type of protection that is available to all users and is located directly on the printing product. They are identified organoleptically, without the use of special equipment. The second group, certified protections, is a complex of protections about which only a specific group of interested parties is informed. Information on the type of such protections and methods of their identification is specified in the appropriate certificate and transferred as a trade secret. The last group of protections, hidden protections, is a complex of protections that the manufacturer uses and does not transfer to the organizer of product turnover. This type of protection can only be identified professionally in special laboratories or certification centers. In this case, the possibility of detection and falsification is eliminated. In the case of some paper materials, e.g., documents requiring the highest level of security, various types of protections from all groups are used, in total even several dozen protections [1,2,3].

The most commonly used types of paper security are the fiber composition of the paper, watermarks, security threads, colored security fibers, sequins, glitter, chemical security, laser engraving, and paper marbling. In addition, various types of drawing security, security in the paint applied to the paper, and security in the paper printing process, as well as optical and biometric security, are also used [1,2,3,4,5,6,7,8].

One of the very interesting types of paper security is the application of photochromic substances dissolved in a medium, which change color as a result of UV radiation. This is caused by chemical changes between two isomers of the photochromic substance with different absorption spectra. The changes can be reversible (returning to the original color after removal from the light source) or irreversible [9,10,11]. The use of this type of photochromic effect for marking paper materials is not widely described in the literature, even though it has a very large potential. The existing works describe the use of the following: (i) photochromic inks in the form of micro-sized crystalline powders dispersed in a matrix [9] and (ii) luminescent resin-based inks [11], which, after printing on a paper substrate, change color as a result of UV radiation. In both cases, it was shown that the developed systems change color reversibly, but the possibility of measuring the absorbed dose and the UV dose distribution in 2D was not demonstrated. For this reason, this work was undertaken to produce, characterize, and present the application possibilities, including the dose measurements of photochromic sensors printed on paper.

The aim of this work was to develop reversible, reusable photochromic sensors on a paper substrate with the screen printing method. The assumption was to produce the sensors in a simple, fast, and cheap way and also to make their indications easy to read. The sensors were characterized in terms of color change depending on the absorbed UV radiation dose, chemical analysis using Raman Spectroscopy, and assessment of the morphology and structure of the samples using Scanning Electron Microscopy (SEM) and Micro-Computed Tomography (micro-CT), respectively. The paper also presents the application possibilities of the sensors and methods for their reading. Such a development can be used as an overt or certified marking of paper products for various applications, e.g., documents, securities, parcels, packaging of food products, medicines, cosmetic products, etc.

## 2. Materials and Methods

### 2.1. Preparation of Photochromic Sensors

Photochromic sensors were prepared by the screen printing method on a paper substrate (A4 format, thickness 0.38 mm, grammage 120 g/m^2^, white color, POLspeed, Kwidzyn, Poland), using a printing paste with a photochromic pigment. Printing was performed using a screen EX 63-063/160 PW (NBC, Tokyo, Japan) with the following parameters: 63 mesh/cm, thread diameter 63 µm, color of mesh: white, tension: 18 N/cm. A printing paste containing 70% *w*/*w* water, 5% *w*/*w* thickener (Lutexal Hit, BASF, Ludwigshafen, Germany), 15% *w*/*w* binder (Helizarin Binder, BASF, Ludwigshafen, Germany), and 10% *w*/*w* photochromic pigment (PD-magenta, SFXC, Denton Island, UK) was used. After printing, the samples were immediately dried in a dryer (Binder FD23, BINDER GmbH, Tuttlingen, Germany) at 80 °C for 30 min.

To investigate the possibility of delaying the color change reaction of the photochromic pigment, some samples were additionally printed with a layer of printing paste with the addition of 30% *w*/*w* of Rayosan^®^ C retarder (Archroma, Pratteln, Switzerland). The retarder concentration used was recommended by the manufacturer as optimal for limiting exposure to UVA radiation.

### 2.2. Irradiation of Samples

The samples were irradiated with UVA radiation, which constitutes 95% of the UV radiation reaching the Earth’s surface [12]. Irradiation was performed in a UVA crosslinker chamber (UVP, Cambridge, UK; five lamps of type F8T5 Blacklight, 8W, range: 315–400 nm; peak at 369 nm, Hitachi, Tokyo, Japan). The device has a built-in detector and control system, as a result of which the dose specified on the panel is delivered to the sample automatically.

### 2.3. Light Reflectance Measurements

A reflectance spectrophotometer (Spectraflash 300; xenon lamp D65; angle 10°; 10 nm resolution; the measurement error is 0.1%; DataColor, Rotkreuz, Switzerland) was used to register the reflectance spectra, as well as the color changes of the samples after irradiation in the CIE Lab color system according to the standard ISO/CIE 11664-4 [13]. The device was calibrated before the measurements, and the calibration procedure was described in detail elsewhere [14]. All measurements were performed with UV light automatically switched off by the software (microMATCH v. 3.6; DataColor), so as not to affect the reliability of the results by additional sample irradiation. Each sample was automatically measured five times. The microMATCH v. 3.6. software generates averaged measurement values without standard deviation and statistical analysis with an accuracy of two decimal places for each wavelength value, from 400 to 700 nm.

### 2.4. Micro-Computed Tomography Scanning

The structure of the printed paper samples was analyzed using high-resolution X-ray tomography, micro-CT (SkyScan 1272, Bruker Corporation, Kontich, Belgium). The tested samples were scanned using the following parameters: X-ray source voltage 50 kV, X-ray source current 200 µA, pixel size 6.5 µm., a rotation step of 0.2°. The 3D reconstructions of the samples were obtained using NRecon 1.7.4.2 and CTvox 3.3.0 r1403 software (Bruker Corporation, Kontich, Belgium). Geometrical parameters of the tested composites were calculated using CTAn 1.17.7.2+ software (Bruker Corporation, Kontich, Belgium).

Micro-computed tomography enables identification and visualization of individual parts of a composite based on the contrast of different image areas resulting from different absorption values of X-rays by the components of the scanned object due to the difference in density. Namely, the absorption of X-rays by paper is different from the absorption of X-rays by printing paste.

### 2.5. Raman Spectroscopy Measurements 

The chemical structure of the samples was investigated using a Raman spectrometer (inVia Renishaw, Gloucestershire, UK), equipped with a 532 nm laser arranged in backscattering geometry. Raman spectra were collected in the range from 100 to 3200 cm^−1^ with 4 mW laser power, and exposure time ranged from 10 to 40 s. All measurements were carried out at room temperature and in an ambient atmosphere.

### 2.6. Scanning Electron Microscopy Imaging

The morphological structure of the samples was analyzed using a scanning electron microscope (TESCAN VEGA3–EasyProbe, TESCAN Brno, s.r.o., Brno, Czech Republic) with a VEGATG software version 4.2.4.0 (high vacuum mode (SE); accelerating voltage 20 kV) and EDX (Bruker, Billerica, MA, USA) detector. Before measurements, the samples were coated with Au-Pd layers using a Cressington Sputter Coater 108 auto system (Cressington Scientific Instruments Ltd., Watford, UK).

### 2.7. The 2D/3D UVA Dose Distribution Measurements

The samples were irradiated with selected doses of UVA radiation, and immediately after irradiation, they were photographed with an iPhone 13 Pro Max (12 MP sensor, 1.9 µm pixels, 26 mm equivalent f/1.5-aperture lens, sensor-shift OIS, Dual Pixel AF, Apple, Cupertino, CA, USA). Then, the images were processed using the polyGeVero^®^-CT v.1.2 (GeVero Co., Lodz, Poland) and polyGeVero^®^ v.2.0 (GeVero Co., Lodz, Poland) software packages [15] in the following steps: filtering, samples calibration, conversion of results into UVA dose distributions, and creation of 2D/3D UVA dose distribution maps.

## 3. Results and Discussion

### 3.1. UV Dose Response of Samples

**Figure 1:**
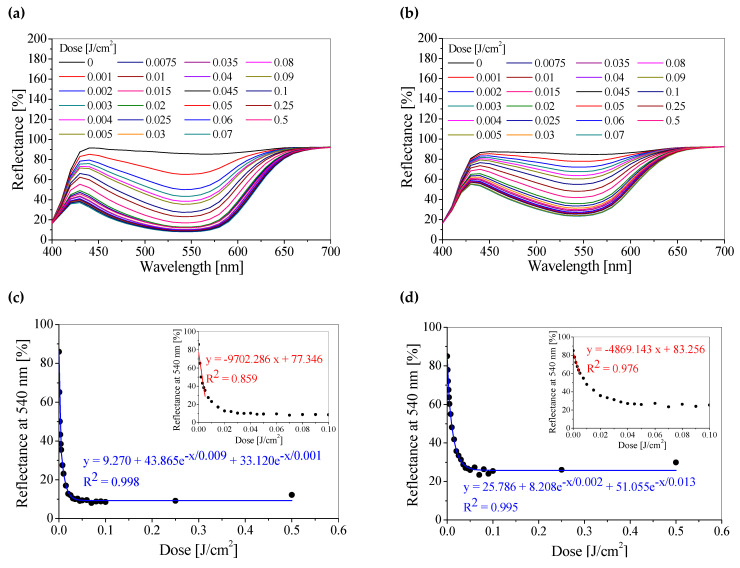
The reflectance spectra of photochromic paper sensors (**a**,**b**) and UVA dose responses of these sensors with fitted curves for the linear (red line, inset) and dynamic (blue line) dose ranges (**c**,**d**). Linear and second-order exponential functions were used, where *y* corresponds to the measured reflectance and *x* to the doses absorbed by the samples. (**a**,**c**) refer to the photochromic sensor without a retarder, and (**b**,**d**) concern the sensor with an additional printed layer of printing paste with a retarder. Insert Figures (**c**,**d**) additionally present the change of light reflectance and calibration equations in the range up to 0.10 J/cm².

The samples were irradiated with different doses of UVA radiation and measured using a reflectance spectrophotometer in the wavelength range of 400–700 nm. As a result of UVA radiation, the samples become pink, and the color is more intense with the higher absorbed radiation dose. This is reflected by the decrease in the light reflectance value with an increase in the absorbed dose in the registered spectra, with a minimum at 540 nm (Figure 1a,b).

In the case of samples covered with an additional layer of printing paste with a retarder, milder color changes are observed with an increase in the absorbed radiation dose, which results from the inhibition of UVA radiation by the retarder. For example, the reflectance value increases by 19.5%, 108.1%, and 197.3% for absorbed doses of 0.001, 0.01, and 0.1 J/cm^2^, respectively. Based on the obtained reflectance spectra, the dose–response relationships of both groups of samples (without and with a retarder) were plotted (Figure 1c,d). A linear range of doses of 0–0.005 J/cm^2^ is observed for both sample groups, and a dynamic range of 0–0.03 J/cm^2^ for the samples without a retarder and 0–0.04 J/cm^2^ for the samples with a retarder. The dose sensitivity, expressed by the slope of the linear regression, is about twice as high for the samples without a retarder compared to the samples with a retarder, which means that the color changes occur with greater dynamics for the samples without a retarder. The color changes of the samples for the selected radiation doses are presented by photographs and color coordinate values in the CIE L*a*b* system (Table 1).

It should be emphasized that the color change is reversible for both samples, without and with a UV retarder layer. After the end of radiation exposure, the pink color begins to fade and changes to the original white color after about 4 min. Reirradiation results in a change to pink. In the conducted studies, such an irradiation cycle was carried out about 200 times, and no changes in the sensor characteristics were observed. The pigment manufacturer ensures that this change is 10,000 cycles. The scope of the conducted studies did not include determining the limit of the color change cycles of the developed sensor.

### 3.2. Morphology of Sensors

**Figure 2:**
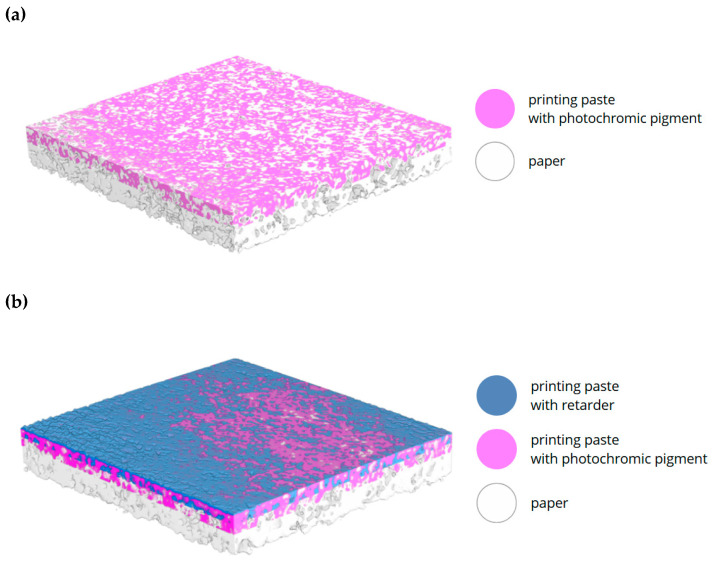
The micro-CT 3D visualization of the photochromic paper sensor without (**a**) and with (**b**) an additional layer of printing paste with a retarder (paper: white color; printing paste with photochromic pigment: pink color; printing paste with a retarder: blue color). The visualizations show samples with an area of 2 × 2 mm^2^.

The subject of structural analysis was a photochromic sensor printed on paper and a photochromic sensor printed on paper and covered with an additional layer of printing paste with a retarder. Figure 2 shows three-dimensional visualizations of both samples made using high-resolution X-ray micro-computed tomography (micro-CT).

Differences in the radiation absorption of the paper, as well as the printing paste with pigment and a retarder, allowed for the identification of each layer of the samples. It can be seen that the photochromic pigment is evenly distributed on the paper surface, which indicates the uniformity of the pigment dispersion in the paste and the uniformity of the print. The paste forms a layer on the paper surface only on one side and does not penetrate the entire volume of the paper (Figure 2a). An additional layer of printing paste with a retarder in some places penetrates the paste with the photochromic pigment (Figure 2b). It should be noted, however, that the paper itself is not perfectly, evenly smooth. There may also be more fillers in some areas, which causes the printing paste to be distributed less evenly. Moreover, a very small area of the sample (2 × 2 mm^2^) was tested, and these places are not visible to the naked eye on the sample after irradiation. The micro-CT analysis indicated that the quantitative ratio of the pigment paste and the retarder paste to the entire volume of the manufactured sensor was 26.6% and 16.8%, respectively.

**Figure 3:**
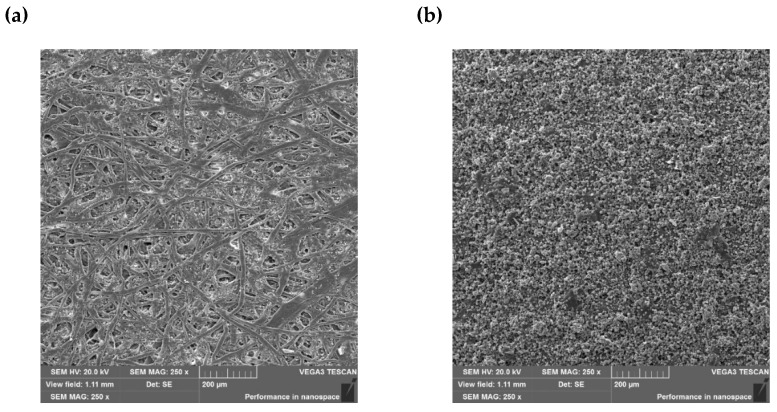

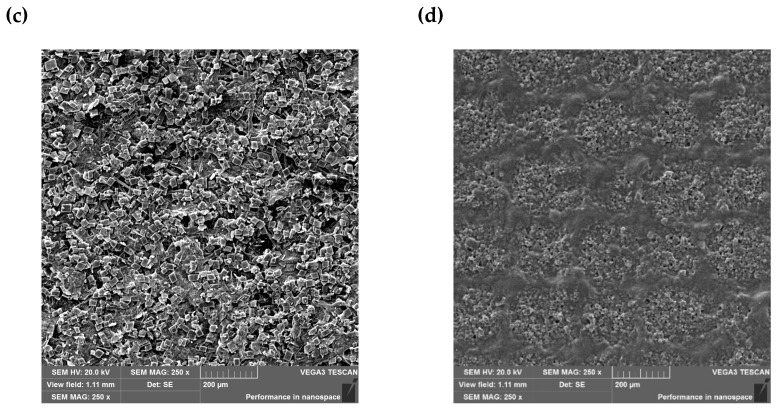
SEM images of paper (**a**), paper printed with printing paste with photochromic pigment (**b**), paper with a retarder (**c**), and paper printed with printing paste with photochromic pigment and with printing paste with a retarder (**d**) at 250× magnification.

The surface morphology of the paper samples was visualized in photographs taken with a scanning electron microscope. Figure 3a shows the structure of the paper with visible cellulose fibers filled with paper-reinforcing substances. After printing the paper with a printing paste containing a photochromic pigment, pigment particles are visible, evenly distributed over the entire surface of the paper (Figure 3b). This indicates the even dispersion of the pigment in the printing paste and the evenly performed print. The particles are round with an average size of 4 ± 2 µm. The additional EDX analysis showed that there are C (67 ± 2%), N (25 ± 2%) and O (8 ± 1%) atoms on the pigment surface. Figure 3c shows small, square, and rectangular particles of the retarder evenly distributed on the paper surface with a regular shape and sharp edges with an average particle size of 23 ± 7 µm. In the case of paper printed with a paste with a photochromic pigment and a paste with a retarder (Figure 3d), a characteristic pattern can be seen on the surface of the samples, which was created as a result of the reflection of the mesh of the printing screen on the printed surface. After printing the paper surface with a second layer of paste, when the screen was lifted, it was locally pulled by the mesh. Thus, at the point of contact between the mesh and the paper surface, areas with greater coverage of the paste with the retarder can be seen. However, it should be emphasized that these areas are relatively small (200 × 200 µm^2^), which does not affect the effectiveness of the retarder.

### 3.3. Raman Spectroscopy Analysis

**Figure 4:**
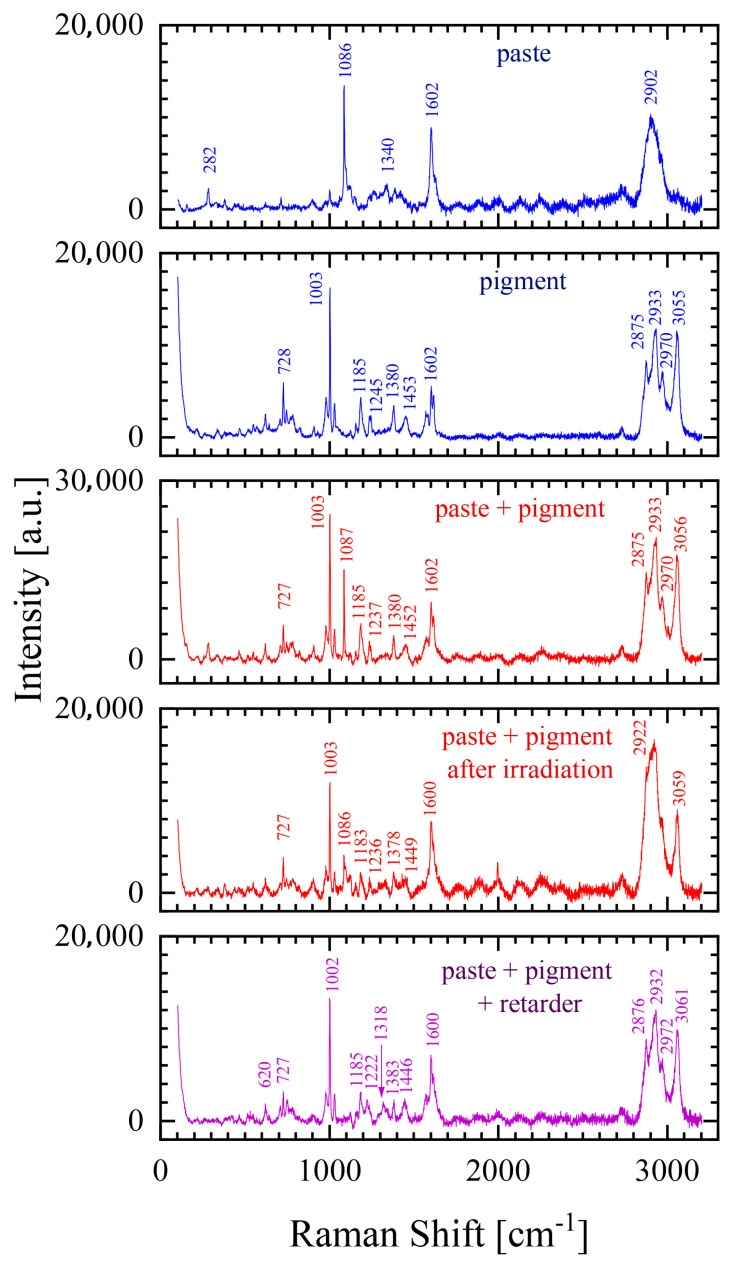
Raman spectra of the photochromic paper sensor and its ingredients.

A photochromic sensor is a complex structure of many components. To clearly describe the changes occurring in its Raman spectrum after the UV radiation process, the Raman spectra of its individual components were also performed (Figure 4). In contrast to those of paper, the Raman spectra of the photochromic sensors and their components are sensitive to the scattering of light at a wavelength of 532 nm (the characteristic bands for paper are clearly visible for scattered laser light at a wavelength of 785 nm), and therefore the Raman spectra of the photochromic sensors and their components do not contain the characteristic bands for the paper substrate [16,17].

The Raman spectra of the photochromic paper sensor and its ingredients (Figure 4) clearly show that the dominant bands of the prepared sensor are the bands for the photochromic pigment. In the region of 2800–3100 cm^−1^, four bands can be identified at 2875, 2933, 2970, and 3055 cm^−1^ characteristic of C-H bonds for the CH_2_ and CH_3_ groups [18,19]. The presence of C-H bonds is also evidenced by the presence in the spectrum of bands at 1453 cm^−1^, the bending of CH_3_ or the bending of C-H naphthalene; and at 1380 cm^−1^, the bending of C-H benzene indoline. And there is also a band at 1087 cm^−1^, the bending of the C-H naphthalene ring, characteristic of the paste. Moreover, in the Raman spectrum of the paste and the photochromic pigment composition, we observe the following: (i) the bands from 1400 to 1650 cm^−1^ are ring modes (the C-C stretching vibrations in the ring), (ii) the band at 1380 cm^−1^ include the C-N stretching vibration present in the spirobenzoxadiazine molecule, and (iii) the bands in the region of 900–1250 cm^−1^ are typically assigned to the C-O stretching vibrations of aromatics [20]. Adding a retarder to this composition does not lead to a change in the nature of the obtained spectrum (Figure 4).

In the above description of the Raman spectrum of photochromic paper, considerable emphasis was placed on the description of the bands characteristic of C-H bonds. This is due to the fact that as a result of delivering a dose of radiation to the photochromic sensor leading to an irreversible change in the sample’s color, these bands are characterized by a significant decrease in intensity (Figure 4). In the case of bands from the region of 2800–3100 cm^−1^, the decrease in intensity is so large that in this region, the band of 2902 cm^−1^ characteristic of the paste begins to dominate and is not visible in the spectrum of the photochromic paper sensor.

### 3.4. Proposition of Applications

**Figure 5:**
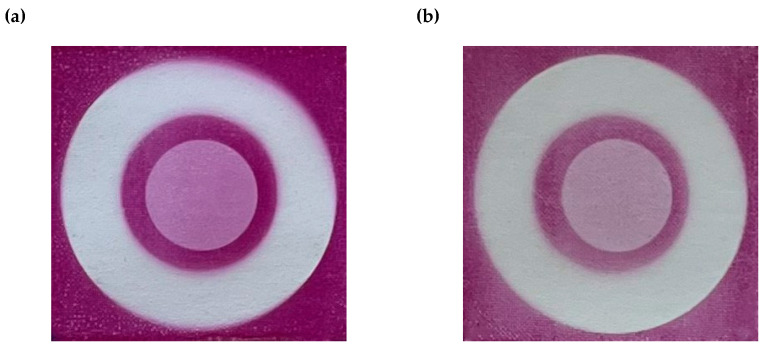
The sensor without a retarder (**a**) and with a retarder (**b**) irradiated non-homogeneously with UVA radiation doses: 0 (white rings), 3 (middle circles), and 30 mJ/cm^2^ (backgrounds).

The developed sensor was analyzed for its ability to measure 2D/3D UV dose distributions. For this aim, the paper sensor samples with and without a retarder with dimensions of 5 × 5 cm^2^ were cut out from printed A4 sheets. For both cases, each sample was irradiated with a different radiation dose in the range of 0–100 mJ/cm^2^. Additionally, to evaluate the 2D/3D dose distribution, the sensor sample with and without a retarder was irradiated non-homogeneously with radiation doses of 0, 3, and 30 mJ/cm^2^ (Figure 5).

**Figure 6:**
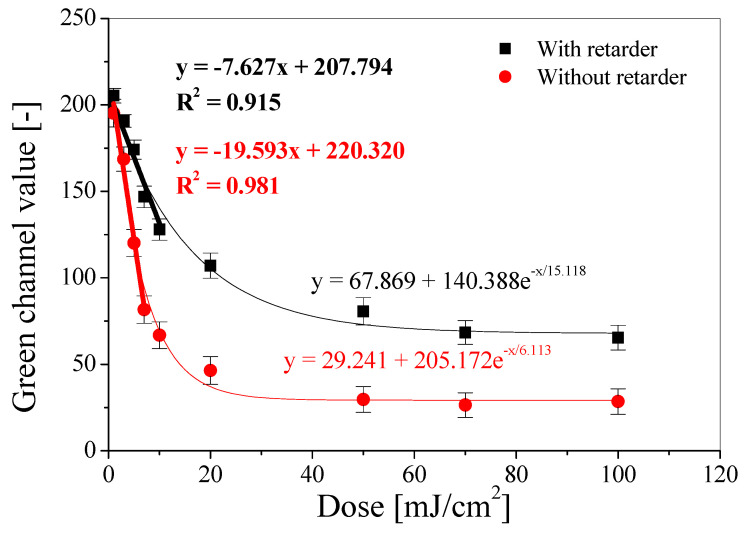
Calibration for the modified paper with (black curves and equations) and without (red curves and equations) a retarder with fitted curves for linear (bold) and dynamic (non-bold) dose ranges.

After irradiation, each sample was immediately photographed using an iPhone 13 Pro Max. The images were then processed in the polyGeVero® v.2.0 software package and decomposed into color channels in the RGB color model. The largest color changes of the samples after irradiation were recorded for the green channel, and for this reason, it was selected for further analysis. The selected settings allowed for the preparation of a calibration relationship between the green channel values and the absorbed UVA dose, which is shown in Figure 6.

Each measurement point is the average value of the entire calibration sample image, an area of 3000 points with marked standard deviation bars. Based on the obtained relationship, it can be seen that the dynamic response of the sensor to the dose is about 30 mJ/cm^2^ for samples without a retarder and about 50 mJ/cm^2^ for samples with a retarder. For higher doses of UVA radiation, no change in dose response is observed. The linear range for samples with and without a retarder is below 10 mJ/cm^2^. The dose sensitivity also decreases with increasing photochromic pigment color conversion. In the case of the samples with a retarder, it can be seen that they are stained more slowly, and the pink color has a lower intensity than for the sample irradiated with the same UVA dose without a retarder. Thus, the sensitivity of the samples with a retarder is 2.5 times higher than for the samples without a retarder. The decrease in the green channel value as a function of the absorbed dose can be described by the calibration equation: (i) for samples with a retarder: y = 67.869 + 140.388e^−x/15.118^, and (ii) for those without a retarder: y = 29.241 + 205.172e^−x/6.113^.

**Figure 7:**
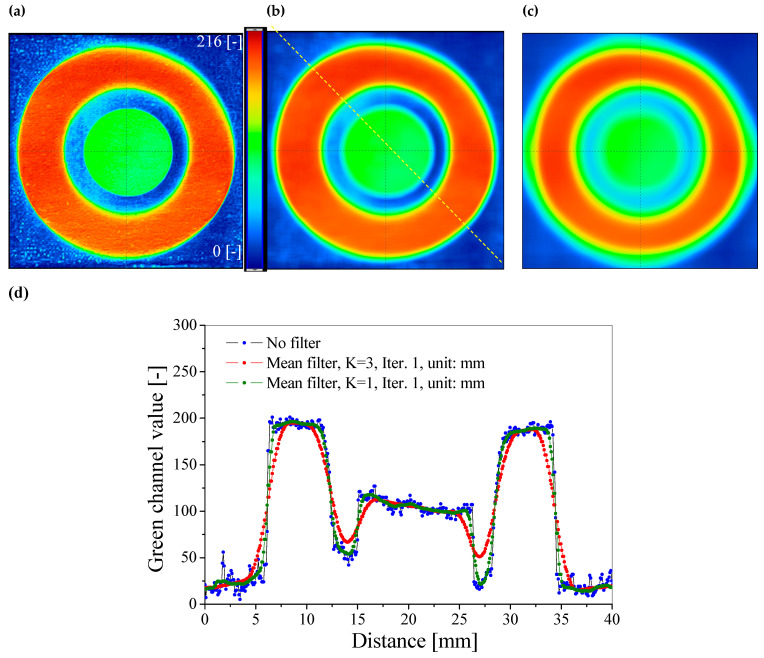
Impact of filtering on image quality. Green channel images of the RGB color model are shown for the sample without a retarder: original sample (**a**), after the mean filter, K = 1, Iter. = 1, unit: mm (**b**), and after the mean filter, K = 3, Iter. = 1, unit: mm (**c**). In (**d**), a comparison of profiles for the images is presented. The profile position is indicated by the yellow dashed line in (**b**).

**Figure 8:**
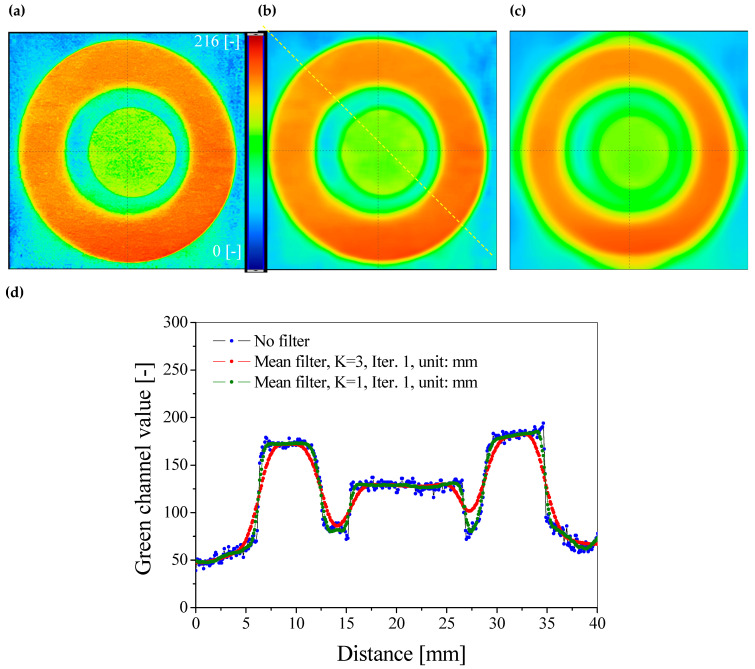
Impact of filtering on image quality. Green channel images of the RGB color model are shown for the sample with a retarder: original sample (**a**), after the mean filter, K = 1, Iter. = 1, unit: mm (**b**), and after the mean filter, K = 3, Iter. = 1, unit: mm (**c**). In (**d**), a comparison of profiles for the images is presented. The profile position is indicated by the yellow dashed line in (**b**).

After scanning, the images were processed without further image manipulations such as filtering and with filtering to reduce the noise corresponding to the sample structure. It should be emphasized that the resulting noise is related to the unevenness of the paper structure, which was also demonstrated in the micro-CT analysis (Section 3.2). The following parameters were used for filtering: the mean filter, kernel size: K = 1 and K = 3, Iteration = 1, unit: mm. The obtained images of the green RGB channels are presented in Figure 7 and Figure 8.

**Figure 9:**
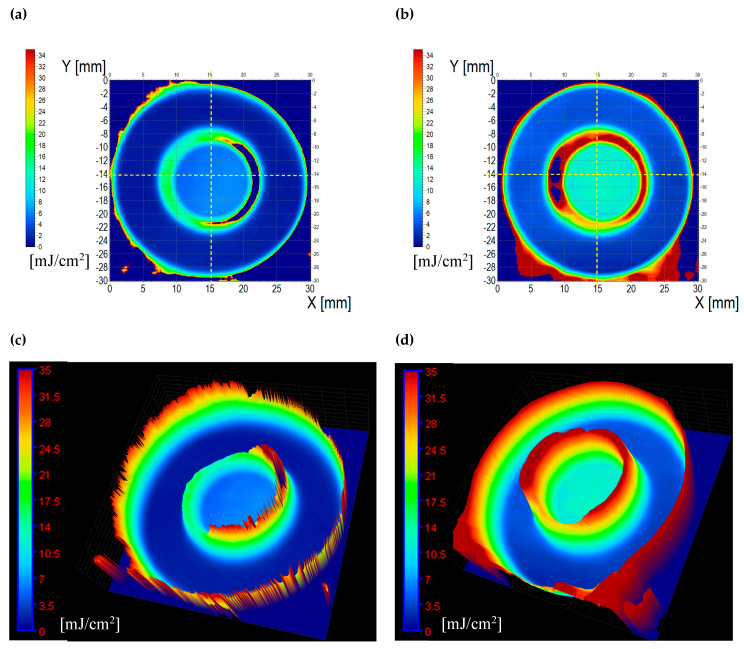
The 2D maps of UVA dose distribution (**a**,**b**) and 3D visualizations (**c**,**d**) for the modified paper samples without a retarder (**a**,**c**) and with a retarder (**b**,**d**).

Filtering with the mean filter of K = 3 was excluded from further data analysis due to too many distorted images obtained after filtering. Appropriate image smoothing and profile shape without changing the dimensions were obtained using the mean filter of K = 1. (Figure 7 and Figure 8). In the next step, the calibration equations were used to generate 2D dose distribution maps of UVA radiation, which are presented in Figure 9a,b. After assigning a color scale and generating 3D maps (Figure 9c,d), the images show areas indicating the registration of UVA radiation doses in the range of 0–35 mJ/cm^2^. It should be emphasized that the range of the linear response of the developed sensors to the dose is up to 10 mJ/cm^2^ (Figure 6), and therefore only doses from this range can be read from the generated maps. After assigning the calibration equations, the dose of 30 mJ/cm^2^ (Figure 5) cannot be precisely read because it is already beyond the linear range.

**Figure 10:**
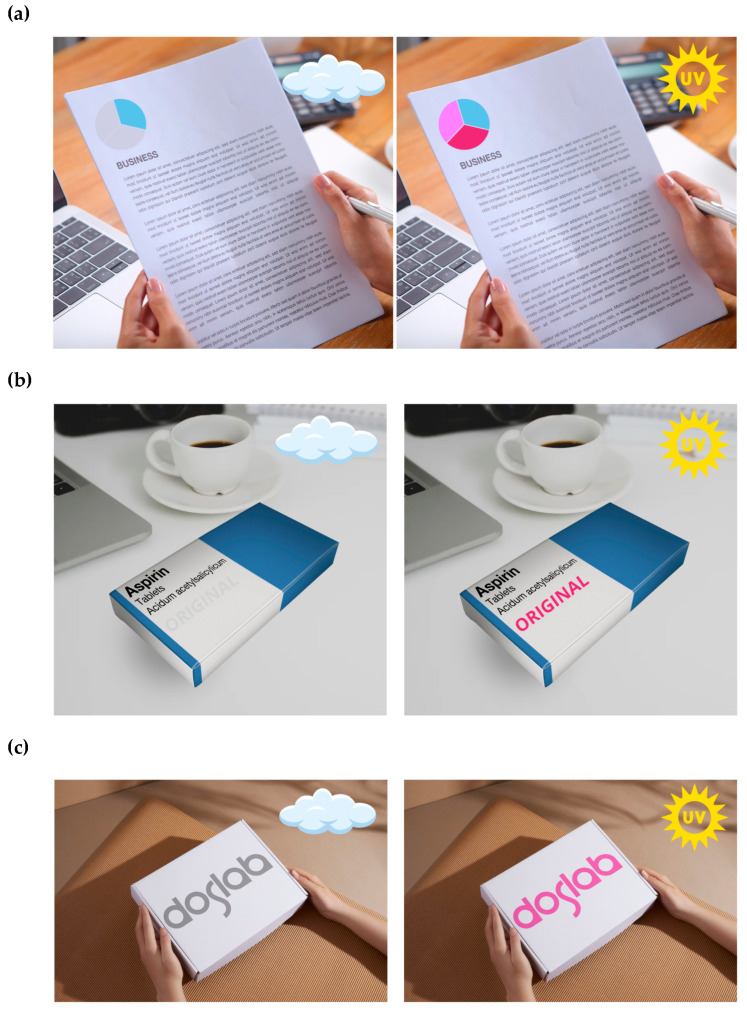
The visualizations of possible applications of photochromic sensors for marking documents (**a**), drug packaging (**b**), and parcels (**c**).

The proposed printing method using photochromic pigments can be also used to mark various types of paper products, including documents, drug packaging, food and cosmetic products, packages, etc. The change in color allows the user to quickly verify, for example, the originality of the product, which can be an element of overt types of security. By designing complex systems, both in terms of the pattern and the selection of the photochromic pigment and retarder concentration, it is also possible to manufacture certified security measures, the verification of which must be consistent with the adopted pattern. Figure 10 presents sample visualizations of possible applications of the developed sensors. The proposed method of manufacturing sensors for securing paper products against counterfeiting is simple, fast, and cheap. Of course, such a security feature can be copied if the exact characteristics of the photochromic pigments used are known. Due to the reversible nature of color changes during exposure to UV radiation and the number of cycles of these changes, we can conclude that the effectiveness of the proposed solution will serve to protect the product during its entire life cycle. In terms of costs, the screen printing method is undoubtedly an efficient and ecological way of functionalizing products. For example, to print a substrate (10 × 10 cm^2^) with a sensor, 0.2 g of paste containing 10% of photochromic pigment is used, and the estimated cost of such a sensor is about 0.02 euro.

## 4. Conclusions

This study presents the development of a photochromic sensor for paper marking. The printing paste containing a photochromic pigment was applied to paper using screen printing, which is a rapid, cost-effective, and simple method. The developed sensor exhibits a reversible color change from white to pink upon exposure to UVA radiation, enabling its multiple uses. The paper included a detailed characterization of the sensor’s color response to UVA radiation. Furthermore, both the structure of the sensor and changes in the sensor’s chemical structure after irradiation were examined. The paper also demonstrated that the proposed manufacturing method ensures uniform distribution of the printing paste with photochromic pigment on the paper surface.

The developed sensor can be used as an overt indicator of paper authenticity, making it suitable for applications such as document security or packaging verification. Additionally, it allows for reading the absorbed dose of UVA radiation, as well as measuring the dose distribution in 2D/3D. A suitable measuring device (reflectance spectrophotometer) or a previously prepared color scale can be used to read the dose recorded by the sensor.

## Figures and Tables

**Table 1:** The color analysis of photochromic paper sensors without and with a retarder, non-irradiated and irradiated with different doses of UVA radiation (0.001, 0.01, and 0.1 J/cm^2^). The photographs were taken with an iPhone 13 Pro Max (12 MP sensor, 1.9 µm pixels, 26 mm equivalent f/1.5-aperture lens, sensor-shift OIS, Dual Pixel AF, Apple, Cupertino, CA, USA) at 23 °C in standard D65 light. The measurement error of Lab values is 0.1%.

Dose [J/cm^2^]	Photograph	CIELab Analysis		
		L*	a*	b*
Photochromic paper sensor				
0	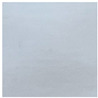	94.69	0.02	0.17
0.001	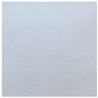	88.06	9.74	−5.34
0.01	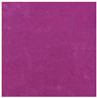	66.71	37.20	−16.73
0.1	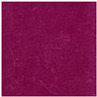	49.70	49.46	−16.21
Photochromic paper sensor with retarder				
0	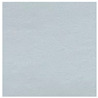	94.22	−0.44	2.50
0.001	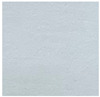	92.11	2.75	0.59
0.01	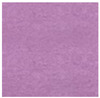	80.87	18.35	−7.38
0.1	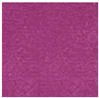	67.48	33.36	−12.52

## Data Availability

The data supporting the reported results are not stored in any publicly archived datasets. The readers can contact the corresponding author for any further clarification of the results obtained.
